# Impact of an Antimicrobial Stewardship Program-bundled initiative utilizing Accelerate Pheno™ system in the management of patients with aerobic Gram-negative bacilli bacteremia

**DOI:** 10.1007/s15010-021-01581-1

**Published:** 2021-02-02

**Authors:** Thomas L. Walsh, Derek N. Bremmer, Matthew A. Moffa, Tamara L. Trienski, Carley Buchanan, Kelly Stefano, Catharine Hand, Tricia Taylor, Karen Kasarda, Nathan R. Shively, Nitin Bhanot, Nicholas Cheronis, Briana E. DiSilvio, Christian Y. Cho, Dustin R. Carr

**Affiliations:** 1 Medicine Institute and Division of Infectious Diseases, Allegheny Health Network, Allegheny General Hospital, 320 East North Ave. East Wing Office Building, Suite 406, Pittsburgh, PA 15212 USA; 2 Department of Pharmacy, Allegheny Health Network, Pittsburgh, PA USA; 3 Department of Microbiology, Allegheny Health Network, Pittsburgh, PA USA; 4 Medicine Institute and Division of Pulmonary and Critical Care Medicine, Allegheny Health Network, Pittsburgh, PA USA

**Keywords:** Rapid diagnostic testing, Accelerate Pheno™, Gram-negative bacteremia, Bloodstream infection, Antimicrobial stewardship

## Abstract

**Purpose:**

Gram-negative bacteria (GNB) are a leading cause of bloodstream infections (BSI) and management is complicated by antibiotic resistance. The Accelerate Pheno™ system (ACC) can provide rapid organism identification and antimicrobial susceptibility testing (AST).

**Methods:**

A retrospective, pre-intervention/post-intervention study was conducted to compare management of non-critically ill patients with GNB BSI before and after implementation of a bundled initiative. This bundled initiative included dissemination of a clinical decision algorithm, ACC testing on all GNB isolated from blood cultures, real-time communication of results to the Antimicrobial Stewardship Program (ASP), and prospective audit with feedback by the ASP. The pre-intervention period was January 2018 through December 2018, and the post-intervention period was May 2019 through February 2020.

**Results:**

Seventy-seven and 129 patients were included in the pre-intervention and post-intervention cohorts, respectively. When compared with the pre-intervention group, the time from Gram stain to AST decreased from 46.1 to 6.9 h (*p* < 0.001), and the time to definitive therapy (TTDT) improved from 32.6 to 10.5 h (*p* < 0.001). Implementation led to shorter median total duration of antibiotic therapy (14.2 vs 9.5 days; *p* < 0.001) and mean hospital length of stay (7.9 vs 5.3 days; *p* = 0.047) without an increase in 30-day readmissions (22.1% vs 14%; *p* = 0.13).

**Conclusion:**

Implementation of an ASP-bundled approach incorporating the ACC aimed at optimizing antibiotic therapy in the management GNB BSI in non-critically ill patients led to reduced TTDT, shorter duration of antibiotic therapy, and shorter hospital length of stay without adversely affecting readmission rates.

## Introduction

Gram-negative bacteria (GNB) are predominant causes of bloodstream infections (BSI), and management is complicated by increasing antibiotic resistance [1–5]. The current standard technique for the diagnosis of BSI is via detection of bacteria from automated blood culture systems and subsequent detection of resistance using agar plates and semi-automatic equipment. This may take 2–4 days, during which time patients may be receiving inappropriate antibiotic therapy. The rapid diagnosis of BSI can improve patient care and foster effective antimicrobial stewardship by allowing optimal targeted therapy to be deployed rapidly [6–8]. The potential benefits of reduced time to effective therapy and time to optimal and definitive therapy include decreased hospital length of stay, reduced mortality, decreased healthcare costs, and reduced downstream effects of unnecessarily broad-spectrum therapy including antibiotic resistance and *Clostridioides difficile* infections [6–11]. Newer rapid diagnostic testing (RDT) can significantly reduce the time to actionable results and allow optimization of therapy within hours of development of sepsis as opposed to a several day delay.

While there are numerous RDTs for GNB BSIs, most of the available technologies focus primarily on pathogen identification (ID). Information on susceptibilities is either unavailable or limited to a few clinically pertinent resistance genes via genotypic testing [8–11]. Detection of resistance genes indicates possible resistant phenotypes to some antibiotics and does not reliably relay information on complete susceptibility profiles and do not provide information for many non-beta-lactam agents. For stable patients with GNB BSI, genotypic testing does not provide susceptibility data for oral antibiotics with excellent bioavailability, such as fluoroquinolones, to promote early intravenous to oral conversion. By focusing exclusively on genotypic resistance, the majority of platforms allows clinicians to escalate therapy, but do not provide data for safe de-escalation to non-beta-lactam oral agents [8–11]. Alternatively, the Accelerate Pheno™ system (ACC) (Accelerate Diagnostics, Tucson, AZ) uses fully automated fluorescent in situ hybridization to provide pathogen ID within 1.5 h and morphokinetic cellular analysis to determine minimum inhibitor concentrations for full antibiotic susceptibility testing (AST) within 7 h (7–8).

While extensive literature exists regarding the impact of RDTs for GNB BSI, most data focus upon microbiologic process measures, such as improvement in time to ID and AST, antimicrobial process measures, such as time to effective therapy (TTET) and time to definitive therapy (TTDT), and outcome measures in critically ill patients managed in intensive care units (ICU), such as mortality (6, 9–11). Conversely, a paucity of data describes the clinical benefits of rapid ID and AST on non-critically ill patients with GNB BSI [12].

To improve management of GNB BSIs, our Antimicrobial Stewardship Program (ASP) developed a syndrome-specific bundled intervention incorporating the ACC. The aims of this intervention were to optimize antibiotic utilization by improving the TTDT and duration of therapy, as well as to shorten hospital length of stay. In this study, we assessed the impact of this ASP intervention for non-critically ill patients with GNB BSIs.

## Methods

### Study setting

Allegheny General Hospital (AGH) is a 631-bed quaternary care teaching facility with approximately 22,000 admissions yearly. West Penn Hospital (WPH) is a 317-bed community-based teaching hospital with nearly 6800 admissions annually. Both facilities are located in Pittsburgh, Pennsylvania and are members of the Allegheny Health Network (AHN). The evaluation was granted exempt status from the AHN Institutional Review Board.

### Study design and population

We conducted a retrospective pre-intervention/post-intervention study comparing the management of non-critically ill patients with GNB isolated from blood cultures before and after implementation of an ASP-bundled initiative. The pre-intervention period was January 2018 through December 2018, and the post-intervention period was May 2019 through February 2020.

### Intervention

In May 2019, the microbiology laboratory at AHN implemented the ACC for all positive blood cultures with GNB identified on Gram stain in patients at AGH and WPH. Prior to the initiation of ACC, our ASP assembled a multidisciplinary task force to create a clinical decision making algorithm for the evaluation and management of GNB BSI. To enhance compliance with the clinical decision making algorithm, we employed a bundled approach:

1. *Dissemination of the clinical decision algorithm* to all medical and house staff via electronic mail
2. *Educational lectures* were presented to Internal Medicine residency house staff, the Department of Hospitalist medicine, the Division of Pulmonary and Critical Care Medicine, and the Division of Infectious Diseases
3. *ACC testing* for all positive blood cultures with GNB identified on Gram stain in patients at AGH and WPH
4. *Real-time communication* of results of Gram stain and AST to the ordering clinician via phone call as well as to the ASP via text page
5. *Prospective audit with real-time intervention and feedback* was performed during the intervention period for all patients with GNB BSI. The ASP contacted primary services via phone call or secure text page to discuss management and provide feedback.

### Microbiology laboratory workflow

Our microbiology laboratory utilized the BD BACTEC™ Automated Blood Culture System (Becton, Dickinson and Company, Franklin Lakes, NJ) for growth and detection of microorganisms present in blood cultures. In the pre-intervention period, when blood culture bottles flagged positive, Gram stain was performed by microbiology technicians and results were called to nurses on the patient’s unit to notify staff of the result. Subsequently, the VITEK^®^ 2 (Biomérieux, Durham, NC,) automated instrument was utilized for ID and AST for GNB during the pre-intervention period, and these results were entered into the electronic health record (EHR) once available. After Gram stain results were communicated to the nursing unit, no further notifications were provided. However, results were communicated to the ASP pharmacists in real time via use of the clinical decision support tool, TheraDoc^®^ (Premier, Charlotte, NC). The ASP team reviewed results Monday through Friday between 7 am and 5 pm, which included prospective audit with feedback to primary teams.

In the post-intervention period, when a blood culture bottle flagged positive, Gram stain was performed by microbiology technicians with results called by technicians to patient’s nurses. If GNB were visualized on Gram stain, secure pages were sent to the ASP pager 24 h per day, 7 days per week. The microbiology technicians’ process of communicating these results to nurses on the patient’s nursing unit continued. Technicians also communicated that ID results would be resulted in 90 min and available in the EHR then. For each isolate, GNB were tested with the ACC in real time. During this time period, a pure subculture on agar continued to be performed with subsequent testing on the VITEK^®^ 2 for ID and AST in parallel. Times for setup, ID, and AST time were recorded. When AST testing by the ACC completed, microbiology technicians again paged the ASP pager to alert that results were available in the EHR and called the nursing unit to inform the care team that AST results were available. These notifications were performed 24 h per day, 7 days per week. The on-call ASP member reviewed the results from the ACC from 7 am to midnight and contacted primary teams with further recommendations.

### Data collection

For the pre-intervention and post-intervention periods, we identified all patients with GNB isolated from one or more blood culture bottles via formal query of our microbiology laboratory database repository and the VITEK^®^ 2 and ACC instruments.

For patients with multiple hospitalizations with GNB BSI, each episode was reviewed. Demographic information, admission and discharge dates, length of hospitalization, patient comorbidities, microbiologic data, radiographic studies, inpatient and outpatient antimicrobial therapy, and subsequent inpatient and outpatient clinical encounters during the 30 days following hospital discharge were collected via review of the EHR, utilizing a standardized data collection instrument. Severity of illness was assessed with the Pitt Bacteremia Score [13]. Dates and times were captured when blood cultures were collected, Gram stain performed, ID, and AST were resulted. Antibiotic utilization during the admission and planned duration as an outpatient were captured for each patient.

Patients were included for analysis if admitted to a non-ICU bed and had a GNB isolated from a blood culture bottle. Patients were excluded for age < 18 years, anaerobic bacteria only isolated, concomitant BSI with *Staphylococcus aureus, Candida* species, or *Enterococcus* species, death within 24 h of positive Gram stain, transferred from an outside hospital when already known to be bacteremic, transferred to an out-of-network hospital during the index hospitalization, received more than 42 days of effective GNB antibiotic therapy, no effective therapy initiated within 48 h of susceptibility data, not admitted to an acute care hospital, planned admission for induction chemotherapy or hematopoietic stem cell transplantation for leukemia, or lack of available data. For the post-intervention cohort, patients were also excluded if ACC was not performed within 8 h of Gram stain revealing GNB.

### Study definitions

The primary outcome was to determine the impact of the ASP-bundle intervention using the ACC on TTDT. Secondary outcomes included the TTET if initial therapy was ineffective, TTDT if initial therapy was ineffective, total duration of antibiotic therapy, duration of intravenous antibiotic therapy, duration of antipseudomonal beta-lactam therapy, hospital length of stay, all-cause and infection-related 30-day readmissions, recurrent infection rates, and formal infectious diseases consultations.

TTDT was defined as the difference between the time that Gram stain was resulted and the administration time of first dose of definitive antibiotic therapy. Definitive antibiotic therapy was defined as the regimen which was utilized by the primary team once AST resulted. TTET was defined as the difference between the time that Gram stain was resulted and the time an antibiotic with in vitro activity was first administered, as defined by Clinical and Laboratory Standards Institute standards. Recurrent infection is defined as a recurrence of bacteremia or primary site infection with the same GNB species within 30 days of initial BSI.

Severe immunodeficiency was defined as use of chronic immunosuppressive therapy at the time of admission (equivalent of > 20 mg prednisone daily), human immunodeficiency virus with CD4 cell count less than 350 cells/mm^3^, active malignancy with receipt of systemic chemotherapy within the 30 days prior to index admission, or receipt of prior solid organ transplant or hematopoietic stem cell transplantation.

### Data analysis

Differences between the pre-intervention and post-intervention cohorts with continuous variables were assessed using the two sample *t* test or Wilcoxon rank-sum test depending on distribution. Differences between categorical variables were assessed using the *χ*^2^ test or Fisher’s exact test as appropriate. *P* < 0.05 was considered statistically significant. Statistical analysis was performed using R, version 3.5.1 (R Foundation for Statistical Computing, Vienna, Austria).

## Results

**Fig. 1:**
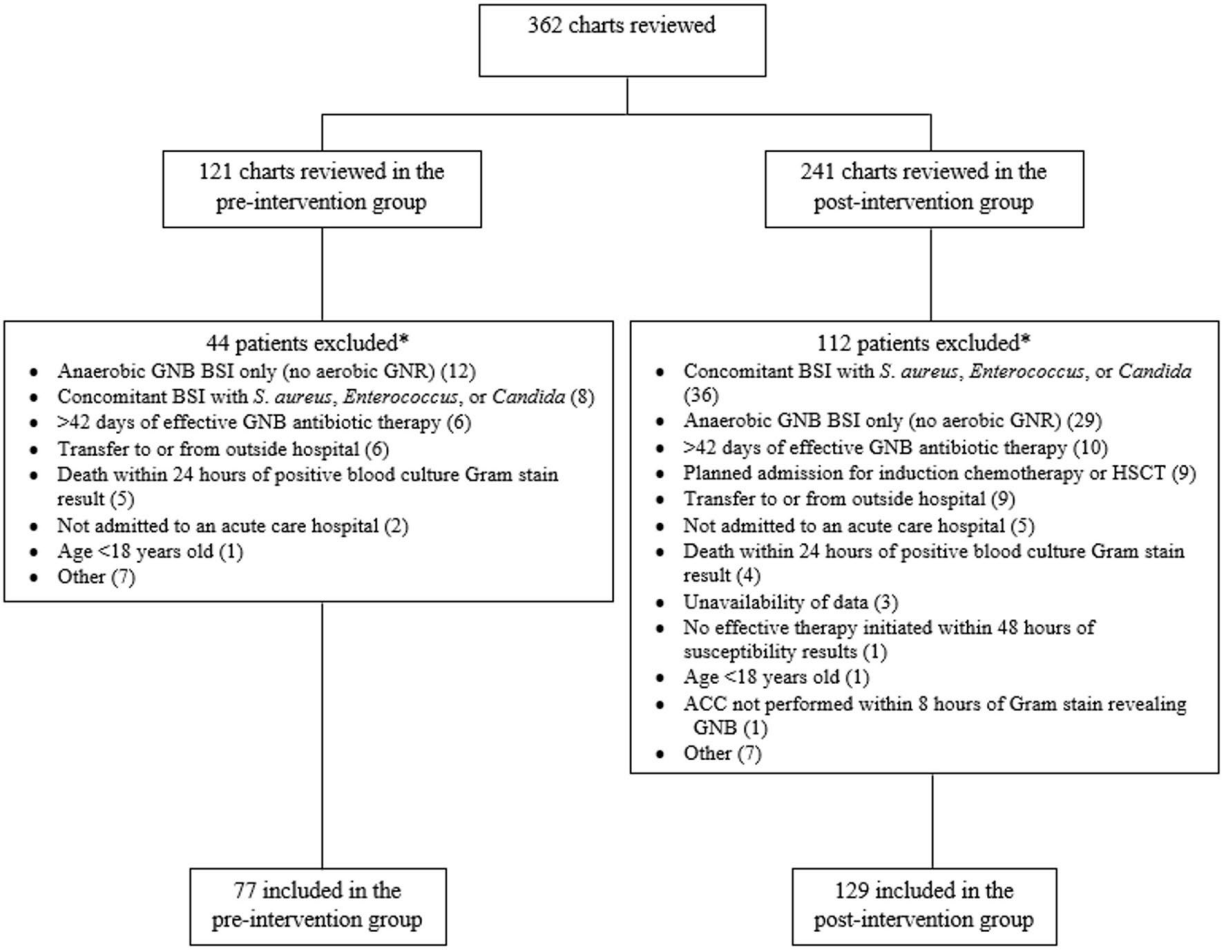
Patient screening. *Multiple exclusion criteria could be applied to each patient. *GNB* Gram-negative bacteria, *BSI* bloodstream infection, *ACC* Accelerate Pheno™ system, *HSCT* hematopoietic stem cell transplantation

During the pre-intervention and post-intervention periods, 121 and 241 patients with GNB BSI were initially identified by microbiology laboratory records, respectively (Fig. 1). After exclusions, the pre-intervention cohort included 77 patients and the post-intervention cohort included 129 patients. Demographic and clinical characteristics were similar except the post-intervention group had more biliary sources (Table 1). The most frequently isolated organisms identified were *E. coli* (42%) and other Enterobacterales (41%). Non-Enterobacterales infection occurred at a rate of 13% with *Pseudomonas aeruginosa* isolated at a rate of 6.3%. Polymicrobial bacteremia occurred equally in both cohorts. Of the 129 patients in the post-intervention cohort, 124 have monomicrobial bacteremia. No ID was given by the ACC in 15 (11.6%) of the 124 with 4 of these being off-panel pathogens. Of the remaining 109 cases of monomicrobial bacteremia, there was concordant ID between the ACC and the VITEK^®^ 2 in 104 (95.4%) and discordance in 5 (4.6%).

**Table 1:** Demographics and clinical characteristics

Characteristic	Pre-intervention (*n* = 77)	Post-intervention (*n* = 129)	*p* value
Age, years^a^	63.7 ± 14.9	66 ± 14	0.28
Female, *n* (%)	42 (54.5)	69 (53.5)	0.88
Race, *n* (%)			
Caucasian	55 (71.4)	97 (75.2)	0.55
African–American	13 (16.9)	31 (24)	0.23
Other	9 (11.7)	1 (0.8)	** < 0.001**
Severe immunodeficiency, *n* (%)	24 (31.2)	41 (31.8)	0.93
Source of bacteremia, *n* (%)			
Genitourinary tract	38 (49.4)	71 (55)	0.43
Catheter related	12 (15.6)	8 (6.2)	**0.03**
Biliary tract	5 (6.5)	26 (20.2)	**0.008**
Intraabdominal	11 (14.3)	13 (10.1)	0.36
Skin and soft tissue	1 (1.3)	5 (3.9)	0.41
Respiratory tract	2 (2.6)	0 (0)	0.14
Other	2 (2.6)	2 (1.6)	0.63
Unknown	6 (7.8)	4 (3.1)	0.18
Pitt Bacteremia Score^b^	1 (0, 2)	2 (1, 3)	0.33
Organism, *n* (%)			
* E. coli*	27 (35.1)	60 (46.5)	0.11
Non-*E. coli* EB	34 (44.2)	51 (39.5)	0.52
* Klebsiella* spp*.*	16 (47.1)	32 (62.7)	
* Enterobacter* spp*.*	6 (17.6)	8 (15.7)	
* Citrobacter* spp.	3 (8.8)	2 (3.9)	
* Proteus* spp.	3 (8.8)	7 (13.7)	
* Morganella morganii*	1 (2.9)	0 (0)	
* Salmonella* spp.	1 (2.9)	1 (2)	
* Serratia marcescens*	4 (11.8)	1 (2)	
Non-EB	13 (16.9)	13 (10.1)	0.16
* Pseudomonas aeruginosa*	4 (30.8)	9 (69.2)	
* Pseudomonas putida*	0 (0)	1 (7.7)	
* Acinetobacter lwoffii*	2 (15.4)	1 (7.7)	
* Stenotrophomonas maltophilia*	3 (23.1)	0 (0)	
* Flavobacterium meningosepticum*	2 (15.4)	0 (0)	
* Vibrio fluvialis*	1 (7.7)	0 (0)	
* Alcaligenes xylosoxidans*	1 (7.7)	0 (0)	
* Moraxella osloensis*	0 (0)	1 (7.7)	
Polymicrobial	3 (3.9)	5 (3.9)	1

^a^Mean ± standard deviation

^b^Median (interquartile range)

*EB *Enterobacterales (isolated organisms include *Klebsiella* spp., *Citrobacter* spp., *Enterobacter* spp., *Proteus* spp., *Morganella morganii*, *Salmonella* spp., *Serratia marcescens*

Bold value indicates p values of < 0.05

The median time from Gram stain to ID decreased from 38.6 to 1.6 h (*p* < 0.001), and median time from Gram stain to AST decreased from 46.1 to 6.9 h (*p* < 0.001). Median TTDT improved from 32.6 to 10.5 h (*p* < 0.001) (Table 2). Nine and 17 patients initially received ineffective therapy in the pre-intervention and post-intervention periods, respectively. In those subgroups, median time to effective therapy improved from 51.2 to 11.2 h (*p* < 0.001).

**Table 2:** Microbiologic outcome data

Variable	Pre-intervention (*n* = 77)	Post-intervention (*n* = 129)	*p* value
Time from Gram stain to organism identification, h^b^	38.6 (26.7, 50)	1.6 (1.5, 1.8)	** < 0.001**
Time from Gram stain to AST, h^b^	46.1 (39.4, 51.9)	6.9 (6.8, 7.3)	** < 0.001**
Time from Gram stain to effective antibiotic therapy, h^a^	− 4.9 ± 22.2	− 8.1 ± 18.2	0.29
Time from Gram stain to definitive antibiotic therapy, h^b^	32.6 (-11, 55.1)	10.5 (-9.4, 22.50)	** < 0.001**
Initial antibiotic therapy ineffective, *n* (%)	9 (11.7)	17 (13.2)	0.76
If initial antibiotic therapy ineffective, time to effective therapy, h^b^	51.2 (43.7, 55.1)	11.2 (10.3, 23.3)	** < 0.001**
If initial antibiotic therapy ineffective, time to definitive therapy, h^b^	51.2 (43.7, 55.1)	11.2 (10.3, 23.3)	** < 0.001**

^a^Mean ± standard deviation

^b^Median (interquartile range)

Bold value indicates p values of < 0.05

**Fig. 2:**
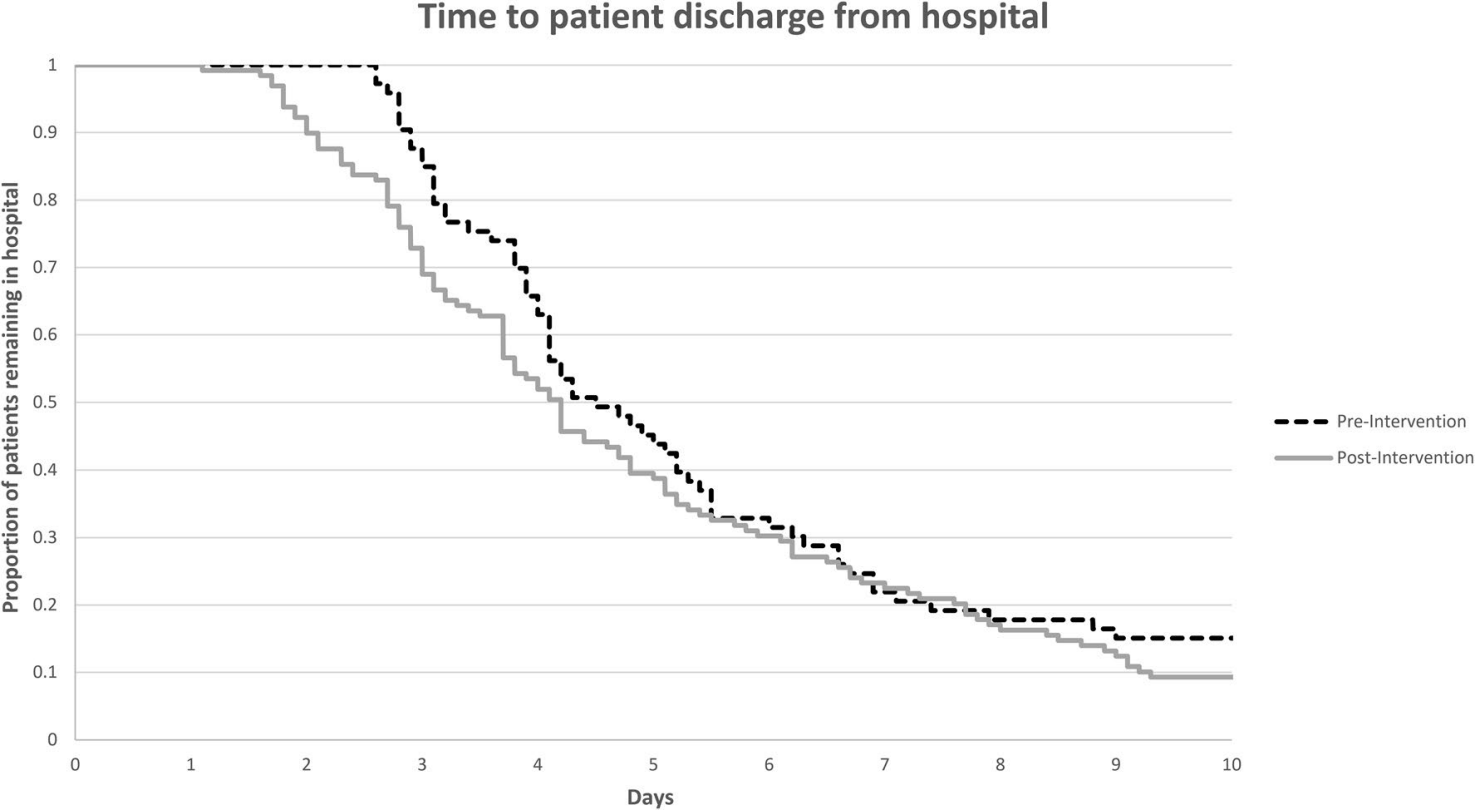
Kaplan–Meier curve

The median duration of total antibiotic therapy decreased from 14.2 days in the pre-intervention cohort to 9.5 days in the post-intervention cohort (*p* < 0.001) (Table 3). More patients in the post-intervention group received less than 10 days of therapy (17% vs 59%: *p* < 0.001). The median duration of IV therapy decreased from 4.5 days in the pre-intervention cohort to 3.8 days (*p* < 0.001). For patients transitioned to oral therapy, median time to oral therapy was decreased from 3.3 days to 2.5 days (*p* < 0.001). For patients with GNB with organisms other than *Pseudomonas aeruginosa, Enterobacter* spp., extended-spectrum beta-lactamase-producing Enterobacteriaceae, and carbapenem-resistant Enterobacteriaceae, median time from Gram stain to antipseudomonal beta-lactam therapy de-escalation decreased from 62.3 h (IQR 54.2, 72.5) in the pre-intervention arm to 35.9 h (IQR 25.5, 43.3; *p* < 0.001) in the post-intervention arm. The mean total hospital length of stay decreased from 7.9 days in the pre-intervention period to 5.3 days (*p* = 0.047) with a significant increase in the percent of patients discharged within 3 days (15.6 vs. 31%; *p* = 0.01) (Table 3, Fig. 2). There was no difference in recurrent infections, all-cause or 30-day readmissions. In the entire study population, the percent of formal infectious disease consultations increased from 51.9 to 79.8% (*p* < 0.001).

**Table 3:** Antibiotic exposure, hospital length of stay, and 30-day readmission

Variable	Pre-intervention (*n* = 77)	Post-intervention (*n* = 129)	*p* value
Total duration of antibiotic therapy, days^a^	14.2 (10.8, 17.8)	9.5 (7.9, 11.2)	** < 0.001**
Total duration of effective antibiotic therapy, days^a^	14.2(10.7, 17.1)	9.3 (7.7, 11.1)	** < 0.001**
Duration of intravenous antibiotic therapy, days^a^	4.5 (3.2, 7.3)	3.8 (2.3, 6)	** < 0.001**
Completed therapy with oral antibiotics, *n* (%)	60 (77.9)	103 (79.8)	0.74
If completed therapy with oral antibiotics, time to oral therapy, days^a^	3.3 (2.4, 4.5)	2.5 (1.7, 3.5)	** < 0.001**
Duration of antipseudomonal beta-lactam, days^a^	2.9 (2, 4.3)	1.9 (1.3, 3.3)	** < 0.001**
Duration of outpatient antibiotic therapy, days^a^	10 (7, 12)	6 (4.5, 7.5)	** < 0.001**
Overall hospital length of stay, days^b^	7.9 ± 11	5.3 ± 3.7	**0.047**
< 3 days, *n* (%)	12 (15.6)	40 (31)	**0.01**
≥ 3 to ≤ 5 days, *n* (%)	32 (41.6)	39 (30.2)	0.10
> 5 to ≤ 7 days, *n* (%)	15 (19.5)	21 (16.6)	0.56
> 7 to ≤ 10 days, *n* (%)	6 (7.8)	17 (13.2)	0.24
> 10 days, *n* (%)	12 (15.6)	12 (9.3)	0.17
All-cause 30-day readmission, *n* (%)	17 (22.1)	18 (14)	0.13
Infection-related 30-day readmission, *n* (%)	3 (3.9)	3 (2.3)	1
Re-infections, *n* (%)^c^	0 (0)	2 (1.6)	0.53

^a^Median (interquartile range)

^b^Mean ± standard deviation

^c^Recurrence of bacteremia or primary site infection with the same Gram-negative bacillus species within 30 days of initial bloodstream infection

Bold value indicates p values of < 0.05

## Discussion

Our ASP-bundled intervention incorporating the ACC to optimize antibiotic utilization in the management of non-critically ill patients with GNB BSI was associated with improved time to ID and AST, TTDT, and shorter duration of antibiotic therapy. Despite decreased antibiotic duration and hospital length of stay, there was no increase in 30-day readmissions or mortality.

Strategies to reduce the utilization of our limited armamentarium of antimicrobial agents are greatly needed in the era of the rise in antimicrobial resistance. Given their significant impact on morbidity and mortality, multidrug-resistant bacteria are considered one of the largest threats to public health [14–17]. While the timing of effective empiric antibiotic therapy in sepsis has shown to be critical, an equally important factor is to limit unnecessary broad-spectrum antibiotics which can lead to the development of resistance [18, 19]. Every additional day of antipseudomonal beta-lactam therapy is associated with an increased risk of new resistance development [18]. Our study incorporating ACC showed the ability to limit antipseudomonal beta-lactam therapy, which has shown to reduce *C. difficile* infection incidence threefold in patients with GNB BSI [19]. Thus, RDTs which provide both ID and AST are invaluable to ensure antibiotic therapy is not only effective but also optimal to maximize clinical cure while minimizing the unintended collateral damage associated with antibiotic exposure.

Limited studies have described a stewardship approach to non-critically ill patients with GNB BSI [12]. Bundling ASP with Biofire^®^ (Biofire Diagnostics, Salt Lake City, UT) blood culture identification decreased time to oral therapy from 5 to 4 days. Interestingly, our analysis showed a decrease to oral conversion from 3.3 to 2.5 days. Since our ASP was already performing prospective audit with feedback, our baseline data with ASP support was similar to the impact that previous studies with ASP have shown. With the addition of ACC, we had additional opportunities to streamline to oral therapy, particularly with fluoroquinolones, that other RDTs could not provide. The rapid availability of fluoroquinolone MICs facilitated earlier discharge on these agents when susceptible in many stable patients who would have previously remained admitted until these MIC data were available.

The Infectious Diseases Society of America, Society for Healthcare Epidemiology of America, and the Centers for Disease Control and Prevention recommend syndrome-specific antimicrobial stewardship interventions to reduce inappropriate antibiotic use [20, 21]. Use of RDTs for GNB BSI represents an ideal ASP target given the frequency of these infections coupled with the prolonged empiric broad-spectrum regimen. ASP involvement is important for the potential benefits of RDTs to be achieved [22]. ASPs are uniquely positioned to collaborate with microbiology laboratories to design workflow to rapidly disseminate results to the primary clinicians, while providing patient specific, real-time guidance to ensure that results are properly interpreted and rapidly acted upon [22]. Indeed, a systematic review and meta-analysis to investigate the impact of molecular RDTs on clinical outcomes in BSI found significantly lower mortality risk with molecular RDT than with conventional microbiology methods, but only in studies with ASP collaboration [6]. In a recent randomized study of patients with GNB BSIs comparing standard of care testing with ASP review or ACC with ASP, rapid ID and phenotypic AST led to quicker antibiotic modification but did not impact patient outcomes including mortality and length of stay [23]. However, this study also included critically ill patients rather than focusing on those with uncomplicated GNB BSIs.

Despite prior evidence demonstrating that patients with uncomplicated GNB BSI can be effectively treated with shorter courses of therapy [24–26], recent evaluations have demonstrated that the duration of antimicrobial therapy for patients with GNB are often prolonged [27–29]. Our intervention was associated with a 33% reduction in duration of total therapy. The 4.7-day reduction in mean duration of therapy for the 129 patients in our intervention cohort equates to 606 fewer days of antibiotics. The education provided during our intervention as well as ASP real-time audit with intervention and feedback contributed to our ability to reduce our duration of therapy.

Our evaluation has several important limitations. First, the retrospective nature of our study design allowed for reviewer bias. Despite chart reviewers not being blinded, we attempted to limit the potential for reviewer bias by utilizing objective endpoints. Second, the retrospective nature of our pre-intervention/post-intervention evaluation lends itself to the potential for period bias. However, there were no other initiatives implemented during this time period aimed at altering hospital antibiotic prescribing practices for GNB BSI. In addition, post-discharge data analysis was limited to readmissions to AGH and WPH. Visits to other inpatient facilities, urgent care centers, and outpatient offices may have been missed, leading to an inability to determine the need to extend or re-introduce antibiotic therapy. We were also unable to assess compliance with outpatient antibiotics. As our inclusion criteria were intentionally selected to only include those patients without critical illness and a need for ICU level support, we cannot comment upon those patients with life-threatening GNB and the ability of an ASP-bundled intervention to impact optimal antibiotic prescribing in this subset of patients. Further study is needed on the impact of ACC with ASP guidance in this patient population. Lastly, our model is a labor-intensive one for an ASP, and this may not be generalizable to smaller ASPs or ones without a similar level of support in regards to dedicated ASP time for physicians and pharmacists to undertake in such an endeavor.

## Conclusions

Our study suggests that implementation of an ASP-guided bundled approach incorporating the ACC represents a practical tactic to enhance antimicrobial use by promoting early de-escalation and reducing duration of both broad spectrum and overall antibiotic duration. Given that our initiative exemplifies an efficacious syndrome-specific strategy for a commonly encountered infection, it is critical to analyze the effectiveness of this bundled approach with rapid ID and AST with real-time intervention from ASP for both critically ill and non-critically ill patients with BSI.

For non-critically ill patients with GNB BSI, an emphasis on early conversion to oral therapy may lead to a reduced hospital length of stay without adversely impacting readmissions or reinfection.
