# Correction to: Impact of an Antimicrobial Stewardship Program-bundled initiative utilizing Accelerate Pheno™ system in the management of patients with aerobic Gram-negative bacilli bacteremia

**DOI:** 10.1007/s15010-021-01601-0

**Published:** 2021-03-17

**Authors:** Thomas L. Walsh, Derek N. Bremmer, Matthew A. Moffa, Tamara L. Trienski, Carley Buchanan, Kelly Stefano, Catharine Hand, Tricia Taylor, Karen Kasarda, Nathan R. Shively, Nitin Bhanot, Nicholas Cheronis, Briana E. DiSilvio, Christian Y. Cho, Dustin R. Carr

**Affiliations:** 1 Medicine Institute and Division of Infectious Diseases, Allegheny Health Network, Allegheny General Hospital, 320 East North Ave. East Wing Office Building, Suite 406, Pittsburgh, PA 15212 USA; 2 Department of Pharmacy, Allegheny Health Network, Pittsburgh, PA USA; 3 Department of Microbiology, Allegheny Health Network, Pittsburgh, PA USA; 4 Medicine Institute and Division of Pulmonary and Critical Care Medicine, Allegheny Health Network, Pittsburgh, PA USA

## Correction to: Infection 10.1007/s15010-021-01581-1

The article “Impact of an Antimicrobial Stewardship Program-bundled initiative utilizing Accelerate Pheno™ system in the management of patients with aerobic Gram-negative bacilli bacteremia” written by Thomas L. Walsh · Derek N. Bremmer · Matthew A. Moffa · Tamara L. Triensk · Carley Buchanan · Kelly Stefano · Catharine Hand · Tricia Taylor · Karen Kasarda · Nathan R. Shively · Nitin Bhanot ·Nicholas Cheronis· Briana E. DiSilvio · Christian Y. Cho · Dustin R. Carr was originally published electronically on the publisher’s internet portal on 02 February 2021 without open access. With the author(s)’ decision to opt for Open Choice the copyright of the article changed on 23 February 2021 to © The Author(s) 2021 and the article is forthwith distributed under a Creative Commons Attribution 4.0 International License, which permits use, sharing, adaptation, distribution and reproduction in any medium or format, as long as you give appropriate credit to the original author(s) and the source, provide a link to the Creative Commons licence, and indicate if changes were made. The images or other third party material in this article are included in the article’s Creative Commons licence, unless indicated otherwise in a credit line to the material. If material is not included in the article's Creative Commons licence and your intended use is not permitted by statutory regulation or exceeds the permitted use, you will need to obtain permission directly from the copyright holder. To view a copy of this licence, visit http://creativecommons.org/licenses/by/4.0/.

